# Environmental Microplastic Exposure Changes Gut Microbiota in Chickens

**DOI:** 10.3390/ani13152503

**Published:** 2023-08-03

**Authors:** Wen Zou, Sijia Lu, Jia Wang, Yixiao Xu, Muhammad Akbar Shahid, Muhammad Usman Saleem, Khalid Mehmood, Kun Li

**Affiliations:** 1 Institute of Traditional Chinese Veterinary Medicine, College of Veterinary Medicine, Nanjing Agricultural University, Nanjing 210095, China; 17119114@njau.edu.cn (W.Z.); 17119116@njau.edu.cn (S.L.); 17119103@njau.edu.cn (J.W.); 9211710527@stu.njau.edu.cn (Y.X.); 2 MOE Joint International Research Laboratory of Animal Health and Food Safety, College of Veterinary Medicine, Nanjing Agricultural University, Nanjing 210095, China; 3 Department of Pathobiology, Faculty of Veterinary Sciences, Bahauddin Zakariya University, Multan 60800, Pakistan; makbar@bzu.edu.pk; 4 Department of Biosciences, Faculty of Veterinary Sciences, Bahauddin Zakariya University, Multan 60800, Pakistan; usmansaleem@bzu.edu.pk; 5 Faculty of Veterinary and Animal Sciences, The Islamia University of Bahawalpur, Bahawalpur 63100, Pakistan; khalid.mehmood@iub.edu.pk

**Keywords:** contaminant, microplastics, gut microbiota, chicken

## Abstract

**Simple Summary:**

The harmful effects of microplastic (MP) exposure on aquatic animals have been extensively studied; however, there is a lack of research on its impact on poultry. To address this gap, the present study aimed to evaluate the effects of MP exposure on the growth performance and gut microbiota of chickens. The findings of the study revealed that MPs had a significant negative impact on the growth performance of chickens and can cause an imbalance in gut microbiota.

**Abstract:**

As novel environmental contaminants, MPs exist widely in the environment and accumulate in organisms, which has become a global ecological problem. MP perturbations of organismal physiology and behavior have been extensively recorded in aquatic animals, but the potential effects of MPs on poultry are not well characterized. Here, we explored the adverse effects of MP exposure on the growth performance and gut microbiota of chickens. Results showed that the growth performance of chickens decreased significantly during MP exposure. Additionally, *Firmicutes*, *Bacteroidota*, and *Proteobacteria* were found to be dominant in the gut microbiota of MP-exposed chickens, regardless of health status. Although the types of dominant bacteria did not change, the abundances of some bacteria and the structure of the gut microbiota changed significantly. Compared with the controls, the alpha diversity of gut microbiota in chickens exposed to MPs showed a significant decrease. The results of comparative analyses of bacteria between groups showed that the levels of 1 phyla (*Proteobacteria*) and 18 genera dramatically decreased, whereas the levels of 1 phyla (*Cyanobacteria*) and 12 genera dramatically increased, during MP exposure. In summary, this study provides evidence that exposure to MPs has a significant impact on the growth performance and gut microbial composition and structure of chickens, leading to a gut microbial imbalance. This may raise widespread public concern about the health threat caused by MP contamination, which is relevant to the maintenance of environmental quality and protection of poultry health.

## 1. Introduction

The production of plastics has increased faster than any other material over the past few decades, and most plastics are eventually released into the environment [1,2]. Statistically, more than half of the plastics produced globally are used in non-recyclable containers, which inevitably cause serious plastic pollution [3]. These plastic products are broken down into MPs through various methods, such as UV-radiation, photodegradation, biodegradation, and mechanical abrasion [4]. MPs are listed as one of the four primary global environmental threats, in parallel with ocean acidification, climate change, and ozone depletion [5]. Land and oceans are the primary habitats of terrestrial and aquatic animals, respectively, and are the ultimate destination for plastics [5,6,7]. Notably, MPs are not only found in natural environments, such as soil, seawater, and freshwater, but are also detected in seafood and beverages, indicating the potential for MPs to be consumed by animals and humans via the food chain [8]. Previous investigations on the hazards of MPs have involved many species and revealed their negative impact on the health of host organisms. For instance, Jin et al. revealed that environmental MP exposure caused gut microbial dysbiosis in mice, accompanied by intestinal barrier dysfunction and disorders of amino acid and bile acid metabolism [9]. Moreover, recent studies on MPs demonstrated that they can lead to hepatic lipid metabolism disorder, kidney damage, and impaired quality of sperm and oocytes [10,11,12]. Although the harm of MP exposure to the environment and organisms has attracted considerable attention, the majority of current research is limited to model and aquatic animals [13]. However, research regarding the effects of MPs on poultry health remains limited [5,8].

As the main ingestion channel for MPs, the gut microbial community will inevitably be affected [14,15]. These microorganisms inhabit the intestines and play a crucial role in host growth and health, because the intestines are the major organs responsible for digestion and absorption [16,17]. Gut microbiota, which are microorganisms residing in the gut including bacteria, viruses, fungi, and protozoa, have been found to play a role in immunity, metabolism, and disease prevention [18,19]. However, gut microbiota is inevitably affected by both host- and environmental-related factors, including smoking, drinking, antibiotics, and host species [20,21,22,23]. In addition to the above-mentioned factors, environmental pollutants are an important factor that perturbs gut microbial homeostasis. Human activities, such as industrial and agricultural production, create a large amount of heavy metals, pesticides, and plastic products every year, which inevitably pollute the environment and threaten animal health [24,25,26,27,28,29]. Previous studies indicated that contaminants such as MPs found in the environment can accumulate in water and plants and can then be transferred to humans and animals by the food chain.

Broiler chickens are a vital source of global meat production [30,31]. Considering the importance of the broiler industry in the global diet, any factors that endanger the health of broilers should be given sufficient attention. However, the health of poultry may be affected by environmental MPs. Previous research reported the presence of MPs in broiler feces, which provided evidence for MP ingestion by poultry [32]. However, studies regarding the influence of environmental MP exposure on growth performance and gut microbial homeostasis in chickens remain scarce. Consequently, we hypothesized that MP exposure may affect the gut microbiota and growth performance of chickens.

## 2. Materials and Methods

### 2.1. Experimental Design

For the animal experiments, a cohort of 60 one-day-old chickens were obtained from a commercial feedlot (Jingzhou, China); these chickens were of similar weight and health status. Standard housing conditions and sufficient diet and water were provided to the chicks to ensure their growth. After three days of acclimatization, the chickens were evenly divided into control (CC) and MP-exposure (MC) groups. The chickens were raised in two cages, with 30 chickens per cage. The control chickens received a normal diet, while the treatment chickens were offered MPs (200 mg/kg) in addition to their normal diet. The MPs provided to chickens were acquired from the Duke Scientific Corporation (product ID CPMS-0.96; Palo Alto, CA, USA); their properties were reported in a previous study [1]. The whole experiment lasted for 28 days, and the dosage of MPs used was based on previous research [33,34]. After the experiment, the chickens were humanely euthanized, and the acquired cecal contents were promptly snap-frozen in liquid nitrogen to preserve their integrity for further analysis.

### 2.2. DNA Extraction and Illumine MiSeq Sequencing

The bacterial DNA was extracted from cecal contents of MC and CC groups using a QIAamp DNA Mini Kit (QIAGEN, Hilden, Germany) based on the manufacturer’s recommendations. Afterward, 0.8% agarose gel electrophoresis and a UV–Vis spectrophotometer (NanoDrop 2000, Waltham, MA, USA) were used to evaluate the integrity and concentration of the extract, respectively. PCR amplification was performed using universal primers (338F: ACTCCTACGGGAGGCAGCA and 806R: GGACTACHVGGGTWTCTAAT) [18,21]. Following the manufacturer’s protocol, the purified products were used to construct sequencing libraries using Illumina TruSeq (Illumina, San Diego, CA, USA). The prepared libraries underwent further processing such as purification, quality control, and fluorescence quantification. The libraries that passed the quality inspection and displayed a single peak were considered qualified. Finally, the qualified libraries were diluted, denatured to single-stranded, and then subjected to 2 × 300 bp paired-end sequencing. To acquire the accurate data in subsequent bioinformatics analysis, the original sequences were preprocessed using QIIME software (Qiime1.9.1, Flagstaff, AZ, USA). Short sequences (<200 bp), mismatched primers, and chimera were removed. The effective reads were then clustered, and operational taxonomic units (OTUs) were partitioned with a 97% similarity threshold. We generated Venn diagrams to distinguish the number and distribution of OTUs in each group. Prior to performing the bacterial diversity analysis, rank abundance and rarefaction curves were constructed to investigate the sequencing depth. We calculated the microbial diversity of chicken gut microbiota by calculating Chao1, ACE, Shannon, and Simpson indices. To investigate the impact of MP on the gut microbiota of chickens, we generated PCoA plots to assess the gut microbial beta diversity. Differential taxa at different levels related to MP exposure were identified using Metastats analysis and LEfSe. The data are presented as mean ± standard error. Statistical significance was determined as a *p* value < 0.05.

## 3. Results

### 3.1. Growth Performance Analysis

**Figure 1:**
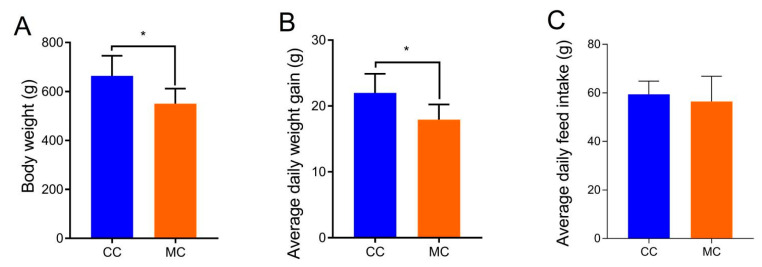
Effects of MPs on growth performance (**A**), average daily weight gain (**B**), and average daily feed intake (**C**) of chickens. All data were represented as means ± SD. * *p* < 0.05.

The body weight and average daily weight gain of chickens in the MC group were significantly lower than those in the CC group (Figure 1), whereas there was no significant difference between the MC and CC groups in average daily feed intake.

### 3.2. Data Analysis

**Figure 2:**
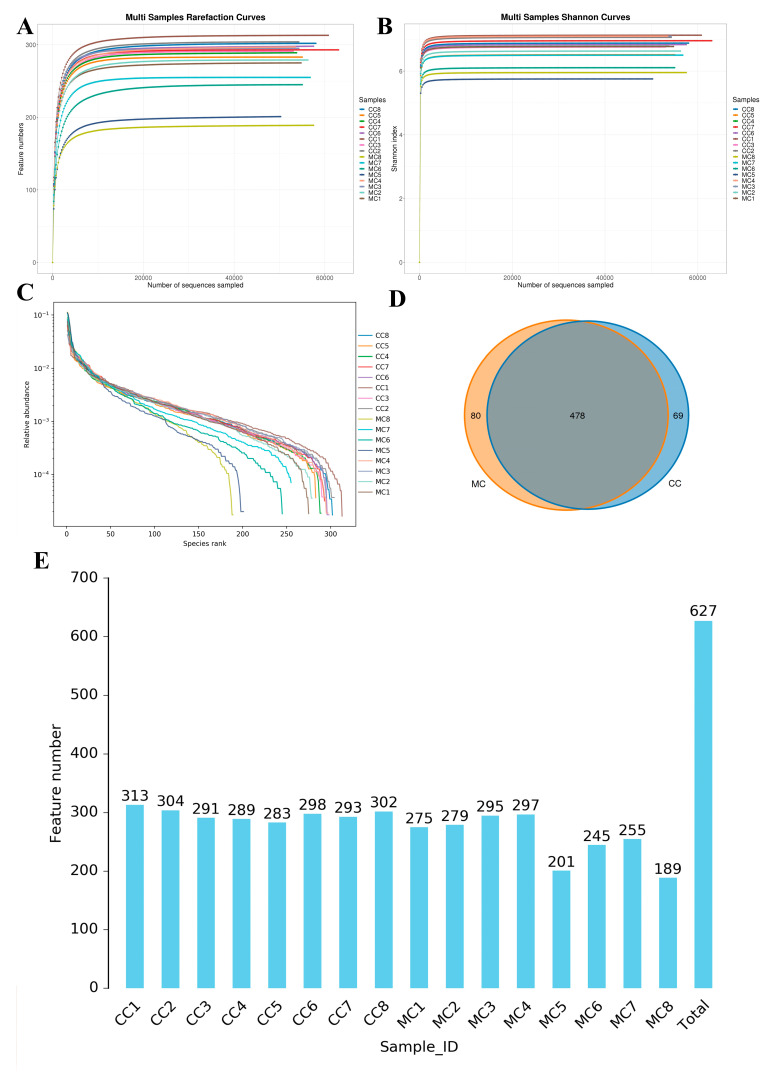
Sequencing depth assessment and OTUs statistics. Sequencing depth and uniformity are assessed by rarefaction (**A**,**B**) and rank (**C**) abundance curves. (**D**): Venn diagram displays the number of shared and individual OTUs in the control and MP-exposed groups. (**E**): Histogram showing the number of OTUs per sample in the control and MP-exposed groups.

In this study, we analyzed 16 cecum samples to compare and investigate changes in the gut microbiota of chickens during MP exposure. We obtained a total of 1,279,763 (CC = 640,238, MC = 639,525) raw sequences, with each sample containing varying raw reads ranging from 79,557 to 80,523 (Table 1). There were 927,938 (CC = 473,198, MC = 454,740) valid sequences in the CC and MC groups after quality evaluation. The rarefaction and rank abundance curves demonstrated a saturation trend, suggesting that further increasing the sequencing depth is unnecessary as almost all bacterial species have already been detected (Figure 2A–C). Following taxonomic assignment, these valid sequences were recognized as 627 (CC = 547, MC = 558) OTUs, with the common OTUs in both the CC and MC groups being 100 (Figure 2D). Furthermore, the numbers of unique OTUs in the CC and MC groups were 69 and 80, respectively. Moreover, the number of OTUs in each sample ranged from 189 to 313 (Figure 2E). Among the samples, CC1 had the highest quantity of OTUs, while MC8 had the lowest.

### 3.3. Significant Changes in the Gut Microbial Diversity Related to MP Exposure

**Figure 3:**
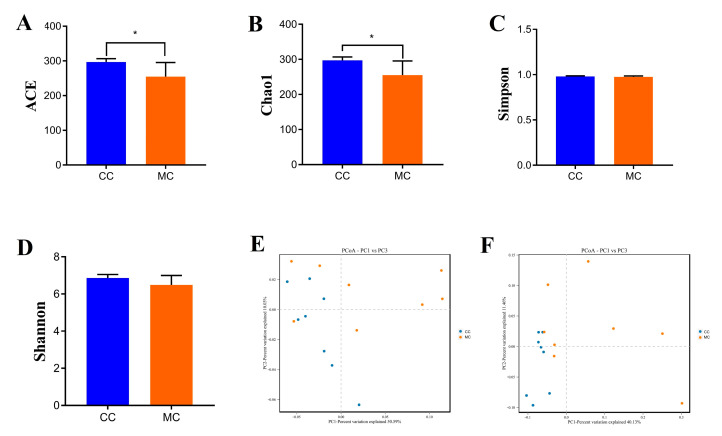
MP exposure altered the alpha and beta diversities of gut microbiota in chickens. Alpha diversity could be represented by the ACE (**A**), Chao1 (**B**), Simpson (**C**), and Shannon (**D**) indices. Beta diversity could be represented by the PCoA scatterplots (**E**,**F**). CC: control group. MC: MP-exposed groups. All data were represented as mean ± SD. * *p* < 0.05.

Good’s coverage estimate in each sample was more than 99%, indicating that almost all bacteria could be covered. In addition, the Chao1 (297.06 ± 9.63 versus 255.06 ± 40.38, *p* = 0.013) and ACE (296.82 ± 9.61 versus 254.89 ± 40.66, *p* = 0.013) indices were significantly different between the CC and MC groups, while the Simpson (0.98 ± 0.0057 versus 0.97 ± 0.010, *p* = 0.20) and Shannon (6.86 ± 0.19 versus 6.48 ± 0.50, *p* = 0.072) indices were not statistically different (Figure 3A–D). The results of alpha diversity analysis showed that the abundance of gut microbiota in chickens decreased significantly during MP exposure, while the diversity of gut microbiota did not show a significant change. Additionally, the samples from both groups were clearly separated, suggesting significant differences in the major components of the gut microbiota (Figure 3E,F). These results demonstrate that MP exposure strongly affects the gut microbial alpha and beta diversities in chickens.

### 3.4. Analysis of Gut Microbiota Composition Associated with MP Exposure

**Figure 4:**
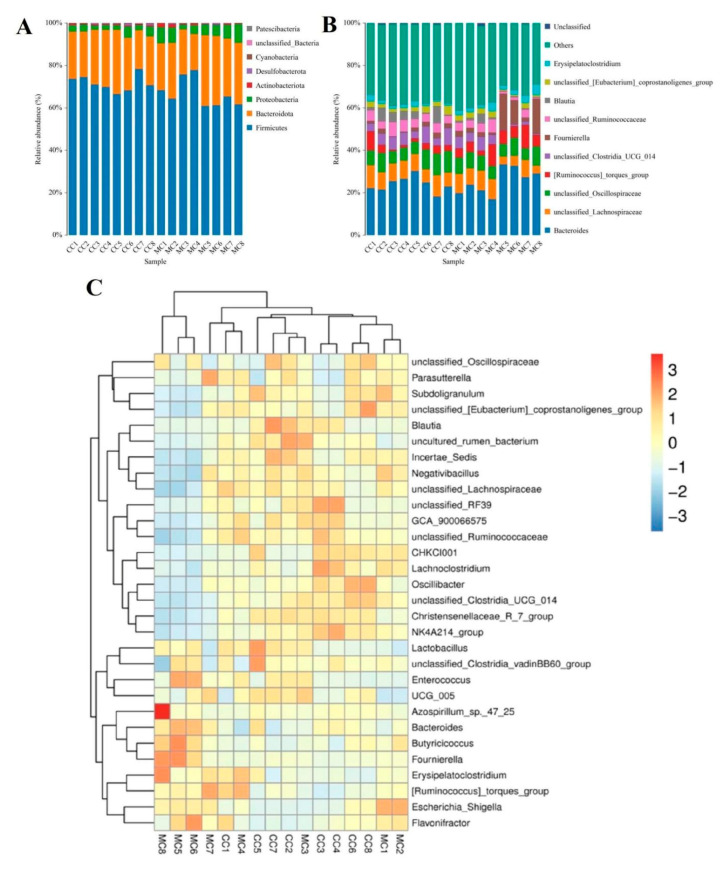
The relative proportions of dominant bacteria in different taxonomical levels. (**A**): Dominant bacterial phyla. (**B**): Dominant bacterial genera. (**C**): The clustering heatmap was used to visualize the distribution and variability of gut microbiota.

To investigate the impact of MP exposure on the gut microbiota, we characterized the compositions and changes of dominant bacterial phyla and genera. Results indicated that a total of 8 phyla and 124 genera were identified, varying from 5 to 8 phyla and from 70 to 99 genera per sample, respectively (Table 2). Specifically, the gut microbiota in CC and MC groups was predominated by *Firmicutes* (71.74% and 66.89%), *Bacteroidota* (23.94% and 26.08%), and *Proteobacteria* (3.17% and 5.90%) in descending order (Figure 4A). These three dominant phyla accounted for approximately 98% of the total bacterial composition. Other phyla such as *Actinobacteriota* (0.47% and 0.77%), *Desulfobacterota* (0.36% and 0.24%), *Cyanobacteria* (0.19% and 0.06%), *unclassified_Bacteria* (0.10% and 0.024%), and *Patescibacteria* (0.0011% and 0.00%) were represented with a lower abundance. Moreover, the dominant genera observed in gut microbiota in the CC group were *Bacteroides* (23.83%), *unclassified_Lachnospiraceae* (8.34%), *unclassified_Oscillospiraceae* (8.03%), and *unclassified_Clostridia_UCG_014* (5.43%), whereas *Bacteroides* (25.46%), *unclassified_Oscillospiraceae* (7.24%), *unclassified_Lachnospiraceae* (6.97%), and *Fournierella* (6.80%) were abundantly present in the MC group (Figure 3B). Additionally, we visualized the clustering heat map to observe the differences in bacterial distribution and variation between the two groups (Figure 4C).

**Figure 5:**
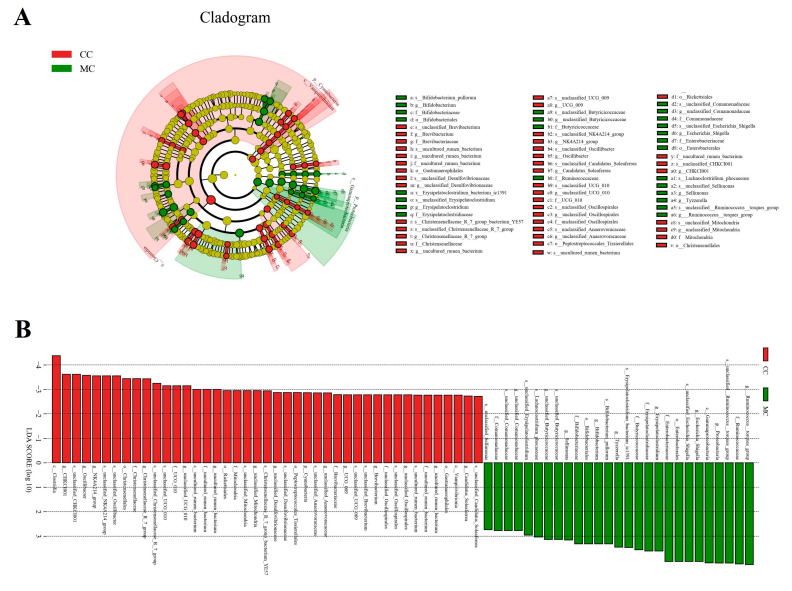
The taxa with significant differences in CC and MC were identified using LEfSe. (**A**): Cladogram showing phylogenetic distribution of differential taxa. (**B**): The criterion of differential taxa was LDA scores > 2.

Metastats analysis was used to identify the differential bacteria at different taxonomic levels between CC and MC groups (Table 3). Compared to the controls, the chickens exposed to MPs showed a significant increase in the abundance of Proteobacteria and a decrease in Cyanobacteria. Moreover, we also found significant changes in 30 bacterial genera with MP exposure. Among them, the relative abundances of 12 genera (*Aerosphaera*, *Facklamia*, *Vagococcus*, *unclassified_Comamonadaceae*, *Bifidobacterium*, *Escherichia_Shigella*, *unclassified_Butyricicoccaceae*, *Sellimonas*, *Tyzzerella*, *Fournierella*, *Butyricicoccus*, and *Ruminococcus_torques_group*) significantly increased. In contrast, the levels of 18 genera (*unclassified_Mitochondria*, *Christensenellaceae_R_7_group*, *unclassified_UCG_010*, *unclassified_Anaerovoracaceae*, *NK4A214_group*, *Jeotgalibaca*, *Novosphingobium*, *Oscillibacter*, *unclassified_Desulfovibrionaceae*, *Blautia*, *Family_XIII_AD3011_group*, *Rikenella*, *unclassified_Oscillospirales*, *UCG_009*, *Brevibacterium*, *unclassified_Clostridia_UCG_014*, *CHKCI001*, and *unclassified_Hydrogenoanaerobacterium*) significantly decreased with exposure to MPs. Notably, MP exposure can lead to the disappearance of some bacterial genera such as *unclassified_Mitochondria*, *Jeotgalibaca*, and *Novosphingobium* in the gut microbiota. Meanwhile, we also used LEfSe analysis to comprehensively identify differential taxa associated with MP exposure (Figure 5A,B). In addition to the differential taxa mentioned above, we also found that *Candidatus_Soleaferrea* and *uncultured_rumen_bacterium* was significantly overrepresented in the CC group, while *Ruminococcus_torques_group* and *Erysipelatoclostridium* were the most preponderant in the MC group.

### 3.5. Correlation Network Analysis

**Figure 6:**
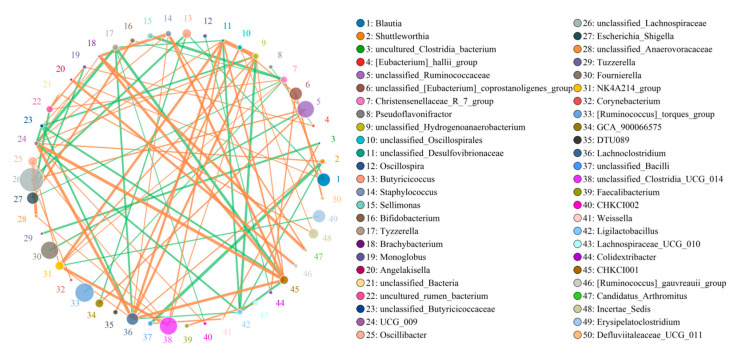
Correlation analysis among bacterial genera. The correlation between bacteria is visualized by the network diagram and the strength of correlation is determined based on the color and thickness of the line. The green line represents a positive correlation, whereas the orange line indicates a negative correlation.

*Blautia* was negatively related to *Butyricicoccus* (0.80) but positively associated with *Christensenellaceae_R_7_group* (0.82) and *Angelakisella* (0.7918) (Figure 6). *Christensenellaceae_R_7_group* was negatively related to *Sellimonas* (0.88) and *Bifidobacterium* (0.78).

## 4. Discussion

The plastic product industry has experienced explosive growth over the past few decades owing to rapid economic development and urban expansion involving many fields of industrial and agricultural production and human life [35]. However, the environmental pollution problems and increased cost of environmental governance caused by the excessive use of plastic products have attracted mounting attention [36,37]. It should be noted that a considerable part of plastic products cannot be recycled but are processed through incineration, deep burial, and discarding which eventually enter the environment and degrade into MPs. The threat of MPs to public health and the health of the animals in husbandry industry has become a prominent issue of concern to many countries and governments. There have been reports on aquatic animals, seabirds, and waterfowl containing MPs, revealing their negative impact on host health [38,39]. The gut microbiota, as the monitor and executor of intestinal function, is inevitably affected by external factors, but information regarding the impacts of MP exposure on gut microbiota in chickens has been scarce. Therefore, we investigated the effects of MP exposure on growth performance and gut microbiota in chickens.

The gut microbiota is naturally stable because of the interaction and plasticity of the microbial community [40]. However, some factors, especially MPs, can disturb the intestinal environment and affect the survival of the microbiota [41]. Under such circumstances, the abundance or type of microorganisms may change to adapt to new intestinal environment, which may lead to the disruption of gut microbial homeostasis. Deng et al. indicated that MP exposure can cause gut microbiota dysbiosis in mice accompanied by metabolic disturbances, increased intestinal permeability, and increased inflammation [42]. Similarly, Sun et al. showed that MP exposure resulted in decreased colonic mucin production, inflammatory responses, and gut microbiota dysbiosis [1]. The indices representing the diversity and abundance include Chao1, ACE, Shannon, and Simpson, which can be used to assess gut microbial homeostasis [43]. Consistent with previous studies, we observed that MP exposure could decrease the Chao1 and ACE indices of gut microbiota in chickens, indicating that MP exposure can decrease gut microbial abundance and induce gut microbial dysbiosis [44]. Maintaining gut microbial homeostasis is crucial for the proper functioning of the intestine, including tasks such as food digestion, nutrient absorption, immune function, and barrier function [45]. However, the perturbation of gut microbial homeostasis may cause various pathological consequences such as intestinal diarrhea, increased intestinal permeability, and metabolic disorders [46,47]. Recent research on gut microbial homeostasis has also revealed its role in the development of diabetes, hypertension, and fatty liver [48]. Therefore, MPs may further cause potential harm to host metabolism, immunity, intestinal function, and health by affecting the homeostasis of gut microbiota. Meanwhile, this may also be one of the reasons for the decreasing growth performance of chickens during exposure to MPs. In addition, we observed significant changes in the major components of the gut microbiota between both the groups. These results demonstrate that gut microbial homeostasis is strongly influenced by MPs.

This study indicated that *Firmicutes*, *Bacteroides*, and *Proteobacteria* were abundant in the gut microbiota of chickens regardless of treatment. These bacteria were demonstrated to be the core components of gut microbiota, which are also abundantly present in ducks, geese, cattle, and pigs [49]. Although the types of dominant phyla did not change, the abundance of some dominant phyla changed dramatically during MP exposure. *Proteobacteria*, composed of a great deal of Gram-negative bacteria, is the largest phylum in the gut microbiota. Remarkably, some members of Proteobacteria were considered as pathogenic bacteria and opportunistic pathogens, which may seriously threaten host health [50]. In this study, the abundance of *Proteobacteria* was significantly increased during MP exposure. Thus, MP exposure may result in an increased risk of intestinal disease and other complications in chickens. Previous investigations indicated that environmental MP exposure could significantly change microbial composition and structure [51]. Similarly, the present research also observed significant shifts in gut microbiota of chickens exposed to MPs. Moreover, some significantly changed taxa were regarded as intestinal functional bacteria, which may play crucial roles in intestinal health and homeostasis. *Christensenellaceae* was considered a potentially beneficial bacterium because of the positive regulation of the hydrolytic enzyme production and intestinal environment [52]. Moreover, *Christensenellaceae* has been demonstrated to be negatively related to metabolic syndrome, inflammatory bowel disease, and fatty deposits [53]. Notably, some quantitatively decreased bacteria such as *Oscillibacter* and *Blautia* were potential producers of short-chain fatty acids (SCFAs). SCFAs have long been deemed as beneficial metabolites due to their vital role in preventing the colonization of pathogens and reducing oxidative stress [54]. Moreover, SCFAs have been shown to possess multiple important biological characteristics such as lowering cholesterol, regulating energy intake, and alleviating inflammation [55,56,57]. Recent investigations on SCFAs also demonstrated their positive impacts in cell proliferation, gut microbial homeostasis, and intestinal barrier function [58,59,60]. Consistent with the current study, MP exposure has also been previously reported to result in a decrease in SCFA-producing bacteria [43]. Importantly, we also found that MP exposure could increase the levels of some pathogenic bacteria, such as *Facklamia* and *Escherichia_Shigella*. *Facklamia* was previously demonstrated to participate in the development of invasive disease such as septicemia and meningitis [61]. *Escherichia_Shigella* is a potentially pathogenic bacterium associated with increased risk of intestinal infections [62]. Moreover, recently published research about *Tyzzerella* also indicated that it could drive the development of cardiovascular disease [63]. These bacteria have been demonstrated to play vital role in the balance of gut microbiota. Thus, we speculated that MPs may further affect gut microbial homeostasis by changing these bacteria.

It is well-established that the gut microbiota is a complex micro-ecosystem involving 10^14^ micro-organisms, approximately ten times the total quantity of body cells [18]. These microorganisms could interact synergistically or antagonistically to maintain gut microbial homeostasis [64]. Consequently, some changed bacteria may directly or indirectly affect the other bacterial functions, thereby further accelerating gut microbial dysbiosis. In this study, we found significant correlations between some bacteria which may be critical for gut homeostasis. This suggests that MP exposure not only directly affects the microbial composition and structure but also indirectly changes the gut microbiota through the microbial interactions, which may further affect gut microbial homeostasis and amplify the toxic effects of MPs.

## 5. Conclusions

In summary, the results of this research support our hypothesis that MP exposure can reduce the growth performance of chickens. Moreover, it also resulted in distinct shifts in gut microbial composition and diversity of chickens. This research is an important exploration of MP exposure on the gut health of farmed animals, suggesting that the imbalance of gut microbiota may be one of the important ways in which MPs lead to ill health.

## Figures and Tables

**Table 1:** Analysis of gut microbial sequence of chickens exposed to MP.

Sample	Raw Reads	Clean Reads	Denoised Reads	Merged Reads	Effective Reads	Effective (%)
CC1	79,984	79,789	79,330	68,122	62,752	78.45
CC2	80,284	80,078	79,731	65,376	56,362	70.20
CC3	79,991	79,797	79,306	66,136	55,469	69.34
CC4	79,872	79,702	79,373	65,742	55,970	70.07
CC5	80,136	79,962	79,522	66,032	57,266	71.46
CC6	79,972	79,800	79,428	67,122	59,480	74.37
CC7	80,023	79,794	79,489	71,109	65,851	82.29
CC8	79,976	79,800	79,445	67,245	60,048	75.08
MC1	79,974	79,785	79,261	66,820	57,389	71.75
MC2	80,113	79,936	79,554	67,864	58,536	73.06
MC3	79,681	79,464	79,109	65,403	56,263	70.61
MC4	79,790	79,601	79,228	64,727	55,667	69.76
MC5	80,005	79,827	79,488	68,398	52,144	65.17
MC6	79,557	79,365	79,029	68,058	56,792	71.38
MC7	80,523	80,306	79,995	68,350	58,891	73.13
MC8	79,882	79,714	79,409	70,800	59,058	73.93

**Table 2:** Species statistics at different taxonomic levels of samples.

Sample	Phylum	Class	Order	Family	Genus
CC1	8	13	34	51	97
CC2	7	12	30	44	91
CC3	7	11	28	45	94
CC4	6	10	28	42	90
CC5	7	12	32	47	93
CC6	7	11	30	44	88
CC7	7	12	29	43	88
CC8	7	12	31	43	92
MC1	5	9	27	44	91
MC2	6	10	28	43	91
MC3	7	12	30	44	90
MC4	7	10	30	50	99
MC5	5	8	24	38	80
MC6	6	10	27	41	88
MC7	5	9	25	42	90
MC8	6	9	25	37	70
Total	8	13	36	61	124

**Table 3:** The bacterial taxa with statistical differences were identified through the Metastats analysis. All the data were represented as mean ± SD.

Taxa	CC (%)	MC (%)	*p*
*Aerosphaera*	0.00 ± 0.00	0.0021 ± 0.0021	0.00099
*Facklamia*	0.00 ± 0.00	0.0023 ± 0.0023	0.00099
*Vagococcus*	0.00 ± 0.00	0.0038 ± 0.0026	0.00099
*unclassified_Comamonadaceae*	0.00 ± 0.00	0.040 ± 0.016	0.00099
*unclassified_Mitochondria*	0.021 ± 0.0052	0.00 ± 0.00	0.00099
*Bifidobacterium*	0.076 ± 0.018	0.48 ± 0.18	0.003
*Christensenellaceae_R_7_group*	0.84 ± 0.043	0.30 ± 0.10	0.003
*unclassified_UCG_010*	0.43 ± 0.045	0.19 ± 0.050	0.005
*Escherichia_Shigella*	0.79 ± 0.29	3.00 ± 0.58	0.0079
*unclassified_Anaerovoracaceae*	0.15 ± 0.013	0.089 ± 0.012	0.0079
*unclassified_Butyricicoccaceae*	0.028 ± 0.019	0.30 ± 0.114	0.0099
*Sellimonas*	0.29 ± 0.054	0.59 ± 0.10	0.011
*NK4A214_group*	1.38 ± 0.18	0.6 ± 0.18	0.014
*Jeotgalibaca*	0.0016 ± 0.0016	0.00 ± 0.00	0.015
*Novosphingobium*	0.0014 ± 0.0014	0.00 ± 0.00	0.015
*Oscillibacter*	1.54 ± 0.22	0.74 ± 0.15	0.019
*unclassified_Desulfovibrionaceae*	0.11 ± 0.015	0.063 ± 0.012	0.03
*Tyzzerella*	0.18 ± 0.053	0.77 ± 0.24	0.031
*Fournierella*	1.57 ± 0.30	6.86 ± 2.47	0.038
*Blautia*	3.6 ± 0.97	1.06 ± 0.50	0.039
*Butyricicoccus*	0.815 ± 0.11	1.41 ± 0.24	0.039
*Family_XIII_AD3011_group*	0.043 ± 0.0095	0.02 ± 0.0045	0.039
*Rikenella*	0.043 ± 0.0054	0.021 ± 0.0074	0.039
*[Ruminococcus]_torques_group*	3.25 ± 0.92	6.26 ± 1.0	0.041
*unclassified_Oscillospirales*	0.33 ± 0.029	0.21 ± 0.047	0.043
*UCG_009*	0.23 ± 0.027	0.14 ± 0.027	0.046
*Brevibacterium*	0.052 ± 0.016	0.013 ± 0.0076	0.047
*unclassified_Clostridia_UCG_014*	5.46 ± 0.68	3.03 ± 0.87	0.047
*CHKCI001*	1.34 ± 0.26	0.57 ± 0.22	0.049
*unclassified_Hydrogenoanaerobacterium*	0.56 ± 0.073	0.32 ± 0.080	0.049

## Data Availability

The original sequence data were submitted to the Sequence Read Archive (SRA) (NCBI, USA) with the accession no. PRJNA954763.
